# Virome Analysis of Near-Bottom Coastal Water of Lake Baikal

**DOI:** 10.1128/MRA.01241-20

**Published:** 2020-12-03

**Authors:** Tatyana V. Butina, Ivan S. Petrushin, Igor V. Khanaev, Yurij S. Bukin

**Affiliations:** a Limnological Institute, Siberian Branch of the Russian Academy of Sciences, Laboratory of Analytical and Bioorganic Chemistry, Irkutsk, Russia; b Limnological Institute, Siberian Branch of the Russian Academy of Sciences, Laboratory of Genosystematics, Irkutsk, Russia; c Irkutsk State University, Irkutsk, Russia; DOE Joint Genome Institute

## Abstract

In recent years, Lake Baikal has undergone significant changes in the composition of coastal communities associated with the increasing anthropogenic influence and global climate changes. In this context, we carried out metagenomic sequencing of the DNA viral community of an integral near-bottom water sample from the littoral zone of the lake.

## ANNOUNCEMENT

Lake Baikal is the world’s deepest, oldest, and largest by volume freshwater lake (1). It has unique environmental conditions and a great biological diversity of mainly endemic flora and fauna (2). Since 2011, the first signs of eutrophication have been observed in the coastal zone of the lake, including changes in the composition and vertical zonation of benthic algae, the presence of unfavorable microbiological indicators, disease and mass mortality of sponges, and others (3–9). Viruses are an important component of the aquatic biota; therefore, the study of viral diversity is useful for assessing their role and dynamics in processes occurring in the coastal zone of the lake.

A shotgun metagenomic study of the DNA viral community, including sample preparation, sequencing, and bioinformatic analysis, was mainly performed as described in our previous studies (10, 11). Briefly, 10-liter water samples were collected in the southern basin of Lake Baikal (near Bolshiye Koty, Russia, 51.9023 N, 105.1028 E) at depths of 10, 12, and 15 m in May 2018 using lightweight diving equipment and a bathometer. The samples were filtered through 0.2-μm nitrocellulose filters (Sartorius) and pooled. The virus-like particles were concentrated with a tangential flow filtration system and the Vivaspin-20 ultrafiltration device (30 kDa; Sartorius) and treated with DNase I and RNase A (Thermo Fisher Scientific). Viral DNA was extracted with a ZR viral DNA kit (Zymo Research).

The preparation and sequencing of DNA libraries were performed in the Center of Shared Scientific Equipment “Persistence of Microorganisms” (ICIS UB RAS, Russia). The paired-end libraries were prepared using a NEBNext Ultra II FS DNA library prep kit for Illumina (New England Biolabs) according to the manufacturer’s protocol. Sequencing of the libraries was conducted on a MiSeq genome sequencer using a MiSeq reagent kit v. 3 (2 × 300 cycles; Illumina).

The primary processing (quality control and trimming) of the virome data set was performed using the R package ShortReads v. 1.48.0 (12); reads with average quality less than 20 and length less than 200 bp were removed. Taxonomic identification of viral sequences was performed using the BLASTn v. 2.5.0 algorithm (13) against the NCBI RefSeq viral complete genome database (14) as described before (11). The BLASTn v. 2.5.0 parameters used were as follows: cost to open a gap, 2; cost to extend a gap, 1; word size for word finder algorithm, 12; penalty for a nucleotide mismatch, 1; and reward for a nucleotide match, 1. The sequence reads were considered “identified” if they had a relative in the reference database with an E value of ≤10^5^ and a bit score of ≥50. For the functional annotation of viral sequences, we used the local BLASTx v. 2.5.0 application (13) and the Clusters of Orthologous Groups (COG) database (15). The BLASTx v. 2.5.0 parameters used were as follows: cost to open a gap, 6; cost to extend a gap, 2; word size for word finder algorithm, 6; E value, ≤10^5^; and bit score, ≥50.

**FIG 1:**
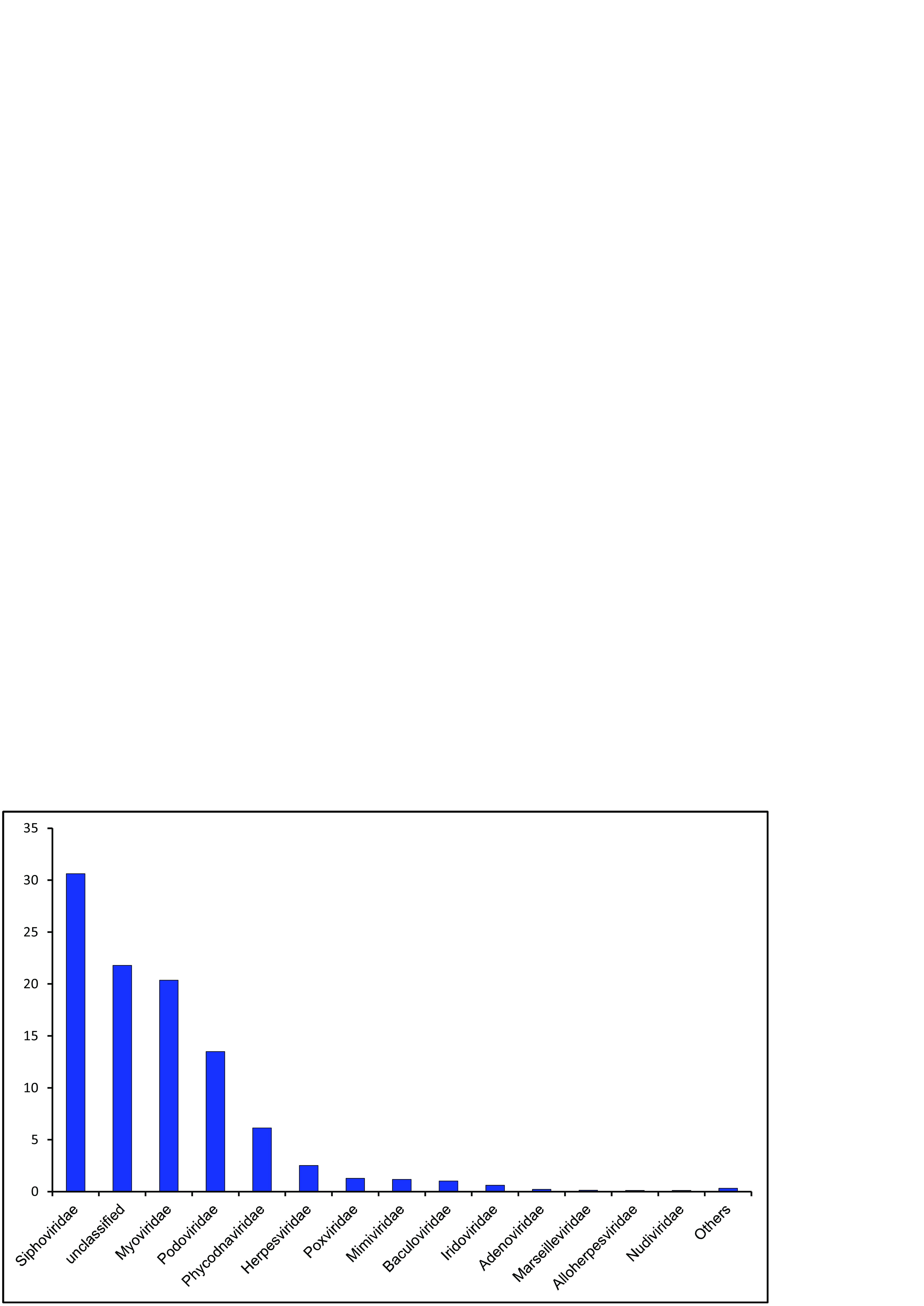
Proportion (percentage) of hits to known viral families in the Lake Baikal virome data set.

The raw data contained 5,329,629 paired sequence reads (or 10,659,258 single reads). After quality processing, we obtained 8,449,571 single reads; of these, 187,941 sequences (2.2% of the data set) were identified as viral, belonging to 26 families of DNA viruses (78.06% of reads), unclassified to the range of family (21.8%), and RNA viruses (0.14%). Thirteen families were the most numerous and together accounted for more than 77% of affiliated virome sequences (Fig. 1). In total, we revealed a high viral diversity in the samples of near-bottom water of Lake Baikal (1,219 virotypes and 22 functional categories of proteins and enzymes).

Our metagenomic research allowed us to describe the taxonomic and functional composition of viral communities in near-bottom coastal waters of Lake Baikal, which will make it possible to assess the role of viruses in the benthic community.

### Data availability.

The raw sequence data have been deposited in the NCBI SRA repository via BioProject PRJNA577390 (BioSample SAMN16330433).
